# Functional genomics in sand fly–derived *Leishmania* promastigotes

**DOI:** 10.1371/journal.pntd.0007288

**Published:** 2019-05-09

**Authors:** Pedro J. Alcolea, Ana Alonso, Ricardo Molina, Maribel Jiménez, Peter J. Myler, Vicente Larraga

**Affiliations:** 1 Department of Cellular and Molecular Biology, Centro de Investigaciones Biológicas (Consejo Superior de Investigaciones Científicas), Madrid, Spain; 2 Center for Global Infectious Disease Research, Seattle Children's Research Institute, Seattle, Washington, United States of America; 3 Laboratorio de Entomología Médica, Laboratorio de Referencia e Investigación en Parasitología, Centro Nacional de Microbiología, Virología e Inmunología Sanitarias, Instituto de Salud Carlos III, Majadahonda, Spain; 4 Department of Global Health, University of Washington, Seattle, Washington, United States of America; 5 Department of Biomedical Informatics and Medical Education, University of Washington, Seattle, Washington, United States of America; National Institutes of Health, UNITED STATES

## Abstract

**Background:**

*Leishmania* development in the sand fly gut leads to highly infective forms called metacyclic promastigotes. This process can be routinely mimicked in culture. Gene expression–profiling studies by transcriptome analysis have been performed with the aim of studying promastigote forms in the sand fly gut, as well as differences between sand fly–and culture-derived promastigotes.

**Findings:**

Transcriptome analysis has revealed the crucial role of the microenvironment in parasite development within the sand fly gut because substantial differences and moderate correlation between the transcriptomes of cultured and sand fly–derived promastigotes have been found. Sand fly–derived metacyclics are more infective than metacyclics in culture. Therefore, some caution should be exercised when using cultured promastigotes, depending on the experimental design. The most remarkable examples are the hydrophilic acidic surface protein/small endoplasmic reticulum protein (*HASP/SHERP*) cluster, the glycoprotein 63 (*gp63*), and autophagy genes, which are up-regulated in sand fly–derived promastigotes compared with cultured promastigotes. Because *HASP/SHERP* genes are up-regulated in nectomonad and metacyclic promastigotes in the sand fly, the encoded proteins are not metacyclic specific. Metacyclic promastigotes are distinguished by morphology and high infectivity. Isolating them from the sand fly gut is not exempt from technical difficulty, because other promastigote forms remain in the gut even 15 days after infection. *Leishmania major* procyclic promastigotes within the sand fly gut up-regulate genes involved in cell cycle regulation and glucose catabolism, whereas metacyclics increase transcript levels of fatty acid biosynthesis and ATP-coupled proton transport genes. Most parasite's signal transduction pathways remain uncharacterized. Future elucidation may improve understanding of parasite development, particularly signaling molecule-encoding genes in sand fly versus culture and between promastigote forms in the sand fly gut.

**Conclusions:**

Transcriptome analysis has been demonstrated to be technically efficacious to study differential gene expression in sand fly gut promastigote forms. Transcript and protein levels are not well correlated in these organisms (approximately 25% quantitative coincidences), especially under stress situations and at differentiation processes. However, transcript and protein levels behave similarly in approximately 60% of cases from a qualitative point of view (increase, decrease, or no variation). Changes in translational efficiency observed in other trypanosomatids strongly suggest that the differences are due to translational regulation and regulation of the steady-state protein levels. The lack of low-input sample strategies does not allow translatome and proteome analysis of sand fly–derived promastigotes so far.

## Introduction: Why is studying sand fly–derived promastigotes important?

The *Leishmania* spp. (Kinetoplastida: Trypanosomatidae) life cycle is digenetic because two hosts are involved: a mammal and a sand fly (being the genera *Phlebotomus* and *Lutzomyia* proven vectors; Psychodidae: Phlebotominae). The promastigote is the motile stage, which develops within the gut of the invertebrate host and is transmitted to the mammalian host during blood sucking (reviewed in [1]). A small fraction of inoculated promastigotes are internalized by mononuclear phagocytic cells [2] and differentiate to the amastigote stage, which is the round, nonmotile dividing form (reviewed in [3, 4]). Eventually, a sand fly feeds from the blood of an infected mammal. Amastigotes are released and transform into promastigotes, which begin the complex developmental process within the sand fly gut, becoming more infective for transmission to the mammalian host [5].

Studying sand fly–derived promastigotes is not exempt from difficulties for three reasons: first, few parasites can be isolated from the insect gut (approximately 2×10^5^ from the whole gut, approximately 10^4^ promastigotes from the stomodeal valve [SV] area) [6, 7] compared with cultures (2–4×10^7^ promastigotes/mL) [8–10]; second, the promastigote populations are phenotypically heterogeneous and asynchronous in the sand fly gut [5, 11–14] and in culture [15]; and third, maintenance of sand fly laboratory colonies, experimental infection, and parasite isolation from the gut are not exempt from technical difficulties [16, 17], being accessible for specialized laboratories. As a consequence, most research on the promastigote stage is performed in axenic culture, and the molecular, biochemical, and physiological features of this stage have been scarcely described within its natural environment. As the genome sequences of these parasites are available [18, 19], high-throughput transcriptome analysis of sand fly–derived promastigotes has been performed in *L*. *infantum* and later on in *L*. *major*.

The main promastigote forms within the sand fly gut are procyclics, nectomonads, leptomonads, and metacyclics [14, 20]. These forms have also been found in culture [21]. The main metacyclic promastigote isolation method is based on the different agglutination ability in the presence of the peanut agglutinin (PNA), despite the structural differences in the lipophosphoglycan (LPG) [22]. Promastigote development in the sand fly gut was extensively reviewed [14, 20, 23].

In vitro infection of the human myeloid U937 cell line with *L*. *infantum* promastigotes showed that the peanut lectin–nonagglutinating metacyclic subpopulation (LiPro-PNA^−^) is more infective than the agglutinating subpopulation (LiPro-PNA^+^) and the whole population in stationary phase of axenic culture (LiPro-Stat), from which both are isolated [24]. The same approach has revealed that LiPro-Stat and LiPro-PNA^−^ are less infective (approximately 50% and approximately 20%–30%, respectively) than promastigotes isolated from the sand fly vector *Phlebotomus perniciosus* (LiPro-Pper) SV [7, 25, 26]. Sand fly metacyclics are present in the SV vicinity, which is located in the thoracic midgut forefront and plays a crucial role in parasite injection into the mammalian host's dermis during blood meal intakes. In the case of the *P*. *perniciosus*–*L*. *infantum* vector–parasite pair, the metacyclic promastigote proportion in culture [24, 25] and within the sand fly gut [27] is approximately equal (approximately 10%). The percentages are much higher (up to 90%) in other parasite and vector species [28, 29]. Culture passage also affects the yield in metacyclic promastigotes [28]. Therefore, higher infectivity levels of sand fly–derived promastigotes isolated from the SV are explained by a more advanced differentiation status (i.e., these promastigotes are more "metacyclic in character") instead of a simple enrichment in metacyclics. Working with promastigotes from the gut is technically demanding, but transcriptome analysis and infection experiments indicate that using the culture model does not always lead to reliable results. Case-by-case decision-making is required in the experimental design [7].

## Promastigote development in the sand fly gut

**Fig 1:**
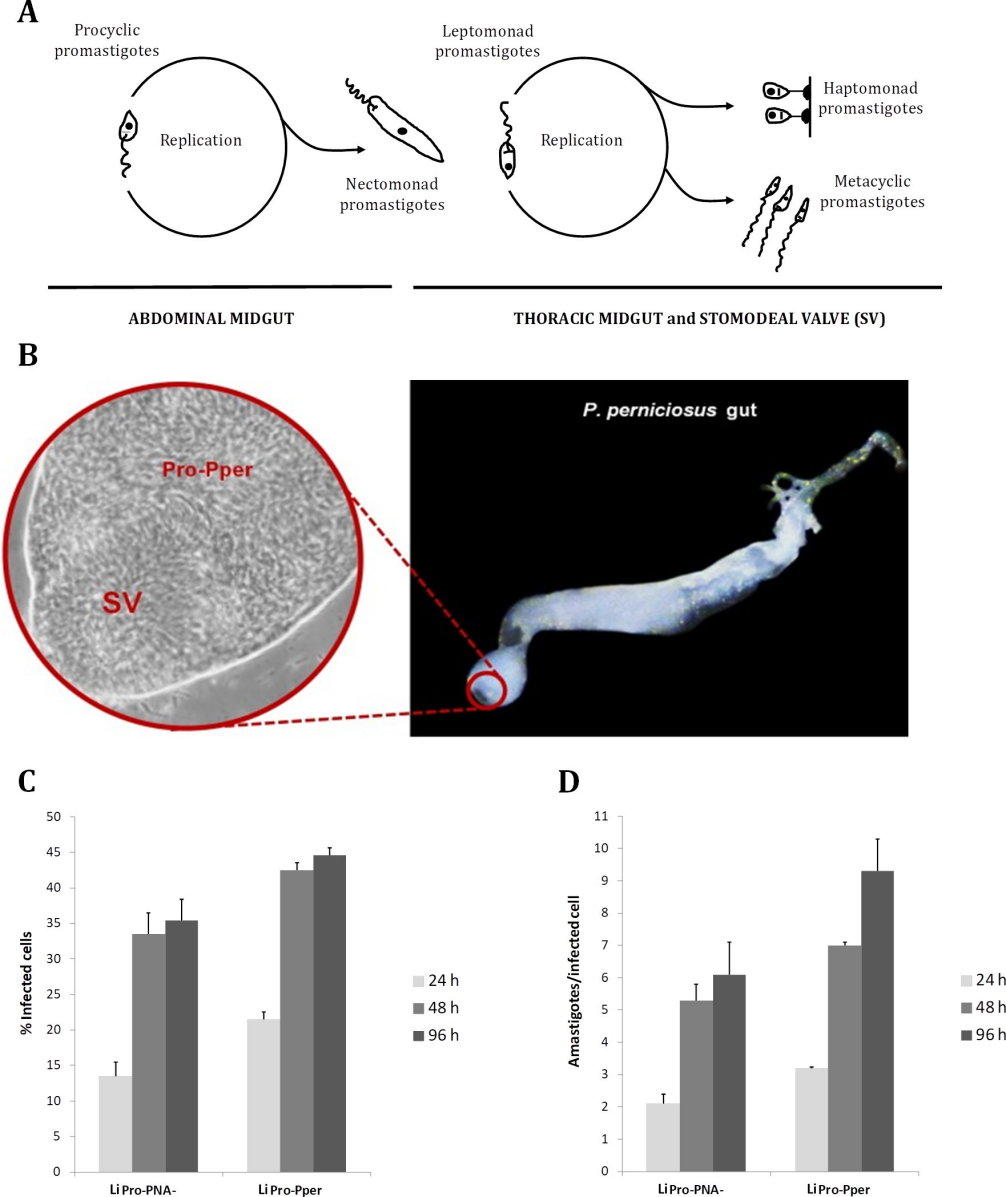
Isolation of metacyclic promastigotes from the sand fly gut. (A) Promastigote stages during development within the sand fly gut. *Adapted from [14]*. (B) Location of metacyclic promastigotes in the anterior pole of the PSG-promastigote plug in contact with the SV. Reproduced from [7]. (C) In vitro infectivity of sand fly–derived *L*. *infantum* metacyclic promastigotes (LiPro-Pper) compared with metacyclic promastigotes from culture (LiPro-PNA^−^) in the human cell line U937. *Reproduced from [25]*. LiPro-PNA^−^, *L*. *infantum* metacyclic promastigotes from culture obtained by the peanut agglutinin negative selection method; LiPro-Pper, *L*. *infantum* metacyclic promastigotes from the *P*. *perniciosus* stomodeal valve; PNA, peanut agglutinin; PSG, promastigote secretory gel; SV, stomodeal valve.

According to Gossage and colleagues’ model [14], based on time course flow cytometry analysis, the *Leishmania* spp. life cycle is completed in three dividing phases, which are separated by nondividing transmission stages. One of them is amastigote replication within mammalian phagocyte phagolysosomes. Then, the blood meal phase takes place in the sand fly abdominal midgut. This phase consists of procyclic promastigote replication followed by differentiation to nectomonad promastigotes. This is valid for suprapylarian species, which are grouped within the subgenus *Leishmania*. Peripylarian species (subgenus *Viannia*) begin development in the hindgut [30]. Nectomonads are nondividing forms with an elongated flagellum that migrate toward the thoracic midgut. During the sugar meal phase, they become leptomonads, which are able to divide. A few leptomonad promastigotes differentiate to metacyclic promastigotes, which are the highly infective stage (Fig 1A). Other forms, like haptomonads and paramastigotes, have been reported. This terminology is useful for understanding development. However, Gossage and colleagues [14] stress the importance of finding molecular markers, which may help in defining these stages more precisely. In *Leishmania* spp., the term “metacyclic” has been defined as the infective form or the end product of promastigote development within the sand fly vector [31], a small rapid-swimming form with an elongated flagellum differentiated from leptomonads [14]. Gossage and colleagues [14] highlighted the absence of parasite–sand fly interactions in axenic culture and warned about improper usage of the terms procyclics and metacyclics when identified with logarithmic and stationary-phase promastigotes, respectively.

Bates [20] and Dostálová and Volf [23] reviewed promastigote–sand fly interactions during development and the hypotheses about the metacyclic promastigotes transmission mechanisms. During the blood meal phase, blood is digested within the chitinous peritrophic matrix (PM), whereas embedded procyclic promastigotes proliferate [32]. Then, nectomonads accumulate in the anterior part of the matrix and are able to escape [33, 34] thanks to the chitinase secreted by the gut epithelium [35, 36]. Nectomonads are able to migrate forward and firmly attach to the gut epithelium microvilli. These facts contribute to explain why the sand fly is a true vector because promastigotes are not expelled during defecation and continue their developmental process. One of the attachment mechanisms in *L*. *major* within *P*. *papatasi* is the LPG interaction with gut epithelium galectins. However, the presence of LPG receptors in other sand fly species remains unclear, and LPG-independent development has been reported. In fact, LPG composition is variable across species. The LPG, together with certain proteophosphoglycans (PPGs), may also have a major role in resistance to proteolysis within the gut (reviewed in [23]). Once nectomonads reach the SV, they become leptomonads and divide [14]. Leptomonads produce the promastigote secretory gel (PSG) [37], mainly composed of filamentous PPG [38], which also lets them bind to the epithelium to some extent. A small fraction of leptomonads become haptomonad promastigotes [39], which tightly attach to the epithelium through hemidesmosome-like structures [40, 41], probably priming PSG plug formation [20] and/or favoring blockage [42, 43], whereas some others differentiate to metacyclic promastigotes [37]. This process is called metacyclogenesis and is defined as the transformation of poorly infective to highly infective promastigotes [28, 44]. In the sand fly gut, metacyclic promastigotes dedifferentiate back into leptomonad-like promastigotes, which have been called retroleptomonad promastigotes, when a second blood meal is ingested by an infected sand fly. Interestingly, retroleptomonad promastigotes rapidly differentiate to metacyclic promastigotes, which causes an important increase in promastigote numbers and infectiousness [29]. Culture passage also causes promastigote dedifferentiation (see "The axenic culture model: Strengths and limitations" section).

According to the blocked fly hypothesis, the PSG plug obstructs the SV until it is removed by regurgitation during blood meal intakes [45]. Leptomonads are embedded, and most metacyclics are located in the plug poles [20]. A different hypothesis is passive inoculation of promastigotes found in the proboscis only [46–48]. Both hypotheses are not mutually exclusive, because both mechanisms may participate in transmission [20]. In fact, low-dose and high-dose bite patterns have been observed and may correlate to the respective aforementioned transmission mechanisms [49]. In addition, chitinase-mediated damage was observed in the SV [33], supporting the regurgitation hypothesis. The pharyngeal and cibarial pumps would contribute to the process [42, 43]. PSG high solubility explains why a few metacyclic promastigotes are released from the PSG plug pole when it contacts blood being ingested (reviewed in [50]). PSG and sand fly saliva egestion accompanying metacyclic promastigotes probably play a role in the initial infection steps [51], including immune response modulation [52–54].

The phenotypical features of the different promastigote forms found in the sand fly gut differ between species. Separately studying each form is challenging. For example, the binding ability is strictly stage dependent, as nectomonads and leptomonads are considerably bound to the epithelium according to the different mechanisms mentioned above and further explained in the next section, whereas procyclics and metacyclics are nonbinding forms. Nonetheless, the relative binding ability is variable between different species, and in certain cases, a mild binding tendency has been observed in procyclics and metacyclics. For example, nectomonads bind tighter than leptomonads in *L*. *infantum*, whereas no substantial differences have been observed in the case of *Leishmania mexicana*, and unlike in *L*. *infantum*, *L*. *mexicana* metacyclics bind slightly tighter than procyclics [55].

## Sand fly–*Leishmania* interactions

Few molecular interactions between *Leishmania* spp. and the sand fly gut have been revealed [23]. The innate immune response to pathogens has been profusely studied in insects, including receptors, signaling pathways, and effectors (antimicrobial peptides, reactive oxygen species [ROS], autophagy, etc.) [56–60]. Defensins, a caspar-like protein, and ROS were associated to innate immunity of the sand fly against *Leishmania* spp. [23, 61–65]. Midgut transcriptomic analysis in *Lutzomyia longipalpis*, *P*. *papatasi*, and *P*. *perniciosus* [66–69] revealed important data about molecules that potentially interact with *Leishmania* spp. molecules.

The blood meal induces digestive enzymes (fundamentally, trypsins and chymotrypsins). These are serine proteases [66–72] like other enzymes induced at the transcript level in the midgut, such as an alanyl aminopeptidase (a novel serine protease), astacin-like metalloproteases, and metallocarboxypeptidases [73]. Resistance to proteases is variable depending on the *Leishmania* species. This feature is crucial for vector competence, defining compatible and noncompatible vectors with a given *Leishmania* species [74–76]. At least half of the amastigote population transforming into immature promastigotes during the first hours of gut colonization are killed, even in compatible species [37]. At the early development stages, the parasite is able to control protease activity levels and timing [66, 72, 77–80] through gene expression modulation and production of serine protease inhibitors (ISPs) in the sand fly midgut when the vector is compatible. The *L*. *major* genome encodes for ISPs that do not have targets in the parasite’s proteome [18] but have been shown to be active against mammalian host phagocytes’ proteases [81] and trypsin activity from sand fly guts [82]. Amastigotes and metacyclic promastigotes are resistant to sand fly gut proteases but not procyclic promastigotes—namely, in the first 2–8 hours of amastigote-to-promastigote transition [83]. Phosphoglycans (PGs) and the secreted acid phosphatase (SAP) are essential for resistance [31]. For example, LPG acts as a shield against proteolytic activities.

The PM is composed of peritrophins, which contain one or more chitin-binding domains (CBDs), which have been predicted in most cases [66, 67, 69]. Multiple CBD peritrophins probably cross-link PM chitin fibrils. PM formation is an extrinsic protection mechanism for promastigotes during blood meal digestion [83, 84]. The sand fly midgut transcriptionally regulates peritrophins in the presence of promastigotes [66, 67]. The PM starts to disintegrate about 2 days after ingestion. A necessary but not sufficient condition for successful promastigote development within the sand fly gut is PM breakage allowing nectomonad promastigote release. This is not always possible depending on parasite and vector species, and parasites’ chitinase implication is controversial [33, 66, 67, 85–88]. Hemoglobin inhibits *Leishmania* spp. chitinase. For this reason, the parasite is not able to escape the PM until blood has been digested [89]. However, chitinases from a given *Leishmania* species are not able to break the PM of all sand fly vector species, and not escaping from the PM leads to parasite elimination through defecation. Therefore, this mechanism contributes to parasite–vector competence [86].

Once nectomonads escape the PM, attachment to the gut epithelium is required to avoid clearance and then progressively ascend throughout the gut. It has been shown that nectomonad and leptomonad promastigotes specifically attach to the gut microvilli, and the mechanism depends on the parasite–vector pair [55, 90]. A molecule involved in attachment is the *Leishmania* spp. FLAG1/SMP1 flagellar protein [91]. According to these interactions, sand fly vectors are classified as restrictive, meaning they are compatible with one or very few *Leishmania* species, and permissive, meaning they support development of multiple *Leishmania* species [92–94]. The most studied parasite–sand fly interaction is the species- and strain-specific *Leishmania* LPG–sand fly midgut galectin attachment mechanism [95, 96]. This interaction has been demonstrated only in the *L*. *major* Friedlin V1 strain–*P*. *papatasi* or *P*. *duboscqi* pairs, but other *L*. *major* strains are not able to bind. The LPG is composed of a glycosylphosphatidyl inositol (GPI) anchor and a glycan backbone composed of PG units and attached to the anchor through a hexasaccharide core [97]. Side-chain composition varies depending on the species and strain [98]. Monogalactosylation is the optimal pattern for galectin recognition, which has been shown through engineered *Leishmania donovani* lines optimized for galactosylation pattern [99]. Also, LPG side-chain composition is stage dependent. Arabinose residues are cap side-chain galactose residues in *L*. *major*, thus allowing promastigote release from galectins [98]. Alternative interaction mechanisms remain undiscovered. Galectins are absent in the midgut of permissive species such as *L*. *longipalpis* and *P*. *perniciosus* [66], which allow survival of LPG-deficient *L*. *major* and *L*. *mexicana* promastigotes in their guts in an LPG-independent manner [23, 45]. However, this is controversial because other authors reported that LPG composition mediates *Leishmania* spp. competence in different vectors [100]. This statement was hypothesized to be valid only in specific vectors [101]. Although LPG-based attachment-release mechanisms in different *Leishmania* spp.–sand fly pairs have been reported, the receptors have not been identified yet (see next section). In summary, it is known that different mechanisms mediate attachment of nectomonad promastigotes to the sand fly gut microvilli, but most remain uncharacterized, and there is controversy about LPG’s roles in different parasite species.

Finally, the sand fly gut conditions may contribute to promastigote differentiation. An acidic environment, nutrient depletion, and probably scarce tetrahydrobiopterin levels induce metacyclogenesis. In this process, endosome sorting and autophagy are essential [102], as well as several *L*. *major* proteins of unknown function encoded in the HASP/SHERP gene cluster (hydrophilic acidic surface proteins and small hydrophilic endoplasmic reticulum proteins) [103].

## The axenic culture model: Strengths and limitations

The first axenic culture of *Leishmania* parasites was performed by Nicolle in the Nicolle–Novy–McNeal medium [104]. Since then, an increasing number of culture media have been developed, leading to easy, fast, and highly productive promastigote cultures. Regarding cell cycle and differentiation, promastigote populations in axenic culture, like in the sand fly gut, are complex and asynchronous. It is assumed that development within the sand fly gut is mimicked in axenic culture at 26–27°C in undefined media containing heat-inactivated mammalian serum [105–110]. Stationary-phase promastigotes are infective despite the absence of parasite–sand fly interactions. However, cultured promastigotes are less infective than metacyclic promastigotes obtained from the sand fly gut, at least in *L*. *infantum* and *L*. *major* [7, 25, 111]. In fact, infectivity is attenuated as the number of culture passages increases. For this reason, passages through laboratory animals are required (reviewed in [109]). These observations highlight the importance of the promastigote–sand fly interactions and suggest that adaptation to the culture conditions results in a progressive loss of the infective properties. Like in the sand fly gut, promastigote populations are heterogeneous in culture, and only a small fraction are metacyclic. The most widespread and successful method to isolate subpopulations of metacyclic promastigotes from cultures is based on LPG agglutination in the presence of the PNA. During metacyclogenesis, the LPG is modified, which leads to the loss of agglutination capability in the presence of PNA [22]. The modifications consist of adding α-D-arabinopyranose residues to the β1,3-D-galactose residue (βGal) side chains [112, 113]. Therefore, the PNA metacyclic selection method is negative. The agglutinating (PNA^+^) subpopulation is less infective than the nonagglutinating (PNA^−^) subpopulation in *L*. *major* and *L*. *infantum* [22, 24]. However, the LPG structure in *L*. *infantum* [114], including *L*. *infantum chagasi* [115], is different and varies depending on the strain, including side chains composed of glucose monomers or oligomers [114]. The LPG of a Sudanese *L*. *donovani* strain agglutinates at early differentiation stages when in contact with PNA [113, 116, 117], but metacyclic forms fail to agglutinate [24, 113, 117–119]. *L*. *infantum* PNA^−^ promastigotes are more infective than PNA^+^ promastigotes [24] and the whole stationary-phase population [25], which suggests that the LPG participates in alternative attachment mechanisms. Soares and colleagues [115] reported an *L*. *infantum* LPG–*L*. *longipalpis* midgut epithelium interaction based on PG receptors. The interaction is based in β1,3-glucosylation, and release is caused by glucose residue removal. The same mechanism was previously described for an Indian *L*. *donovani* strain and the vector *P*. *argentipes* [117]. To add more complexity, the mechanism is opposite in *Leishmania braziliensis* because glucose residue addition leads to ex vivo detachment from *L*. *longipalpis* gut explants [120]. In summary, the LPG–gut interaction and release mechanisms differ between species and are not related to PNA-based separation of procyclics and metacyclics. The minimum agglutinating amount of PNA is variable between *L*. *infantum* strains starting at 50 μg/mL [24, 118]. The different LPG composition in the aforementioned species explains these observations. Interestingly, PNA^−^ and PNA^+^ forms can be isolated in the monogenetic trypanosomatid *Crithidia fasciculata* [121], but the implications for life cycle understanding are unknown.

In vitro infection experiments of the human myeloid U937 cell line with *L*. *infantum* promastigotes have shown that the LiPro-PNA^−^ metacyclic subpopulation is more infective than the agglutinating LiPro-PNA^+^ and the whole population in stationary phase of axenic culture (LiPro-Stat), from which both are isolated [24]. The same approach has revealed that LiPro-Stat and LiPro-PNA^−^ are less infective (approximately 50% and approximately 20%–30%, respectively) than promastigotes isolated from the SV of the sand fly vector *P*. *perniciosus* (LiPro-Pper) [7, 25, 26]. Sand fly metacyclics are found in the SV vicinity. In the case of the *P*. *perniciosus*–*L*. *infantum* vector–parasite pair, the proportion of metacyclic promastigotes in culture [24, 25] and within the sand fly gut [27] is approximately equal (approximately 10%). The percentages are much higher (up to 90%) in other parasite and vector species [28, 29]. Culture passage also affects the yield in metacyclic promastigotes [28]. Therefore, higher infectivity levels of sand fly–derived promastigotes isolated from the SV are explained by a more advanced differentiation status (i.e., these promastigotes are more "metacyclic in character") rather than a simple enrichment in metacyclics.

Considering how challenging working with promastigotes from the gut is, the cost–benefit balance presumably tilts to axenic culture in principle, but this is not as clear when considering results obtained by means of transcriptome analysis. Alternative methods for isolation of metacyclic promastigotes, like centrifugation in Percoll gradient, have been described, which are out of the scope of this review.

## Transcriptome analysis of sand fly–derived promastigotes: Technical considerations and current datasets

**Fig 2:**
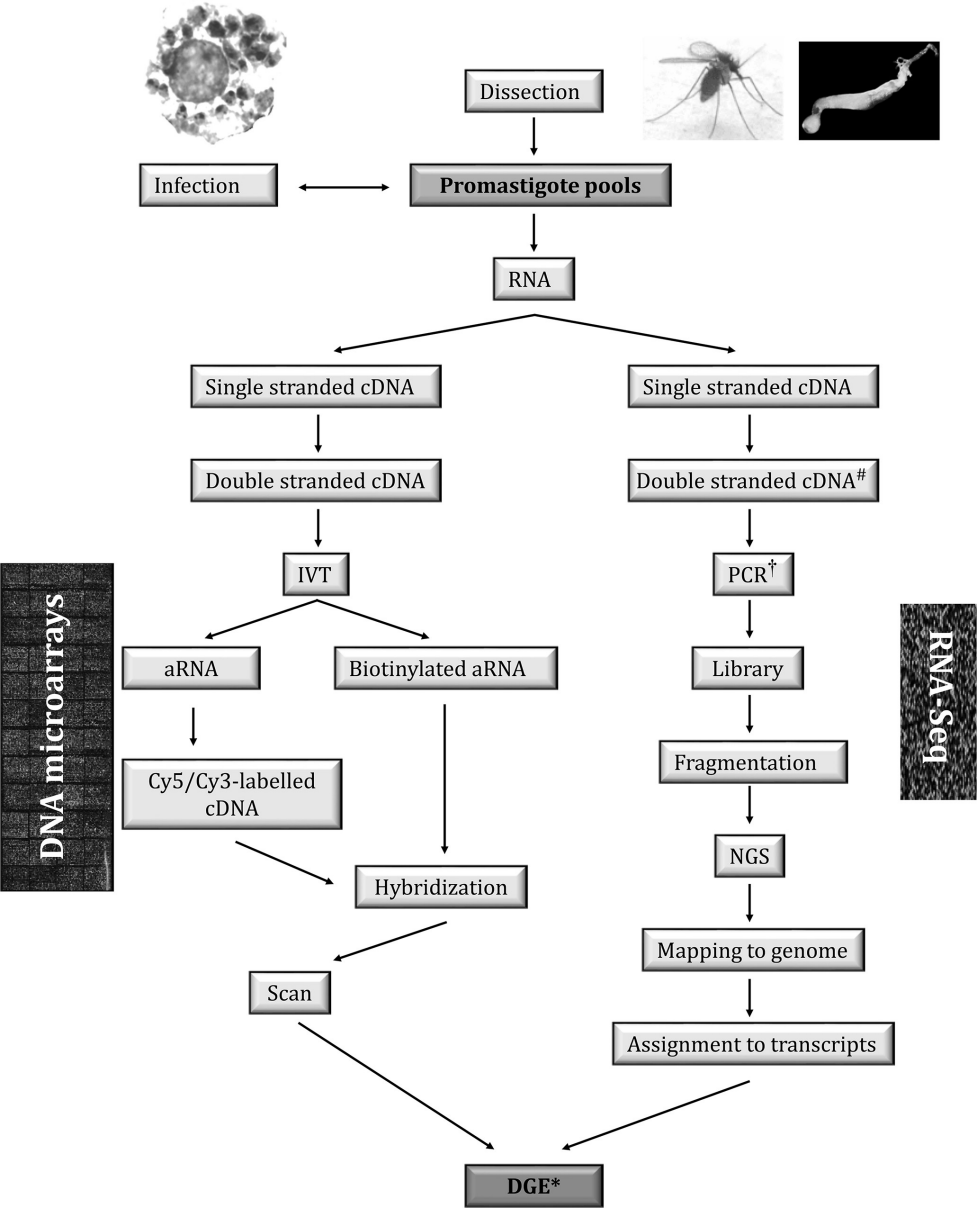
Strategies for DGE analysis of sand fly–derived promastigotes. Only transcriptomics strategies are feasible to date for DGE analysis for very-low-input samples such as sand fly–derived promastigotes. In slRNA-seq strategies, the SL sequence is used in second-strand cDNA synthesis (#), thus increasing specificity when analyzing samples containing genetic material from the host. A cross-hybridization control should be included in microarray experiments to avoid biased results due to noise of the host genetic material. The RNA-seq strategies allow for multiplexed analysis by including indexing sequences during PCR amplification (†). Mapping to genome and alignment to transcript annotations is required during microarray hybridization experiments only when the DNA probes spotted on the slides have not been identified before the experiment (*). An example is shotgun genome DNA microarrays, in which only the clones of interest containing DEGs are sequenced and aligned to identify those genes [24]. aRNA, amplified RNA; Cy3, cyanine 3; Cy5, cyanine 5; DEG, differentially expressed gene; DGE, differential gene expression; IVT, in vitro transcription; NGS, Next Generation Sequencing; RNA-seq, RNA sequencing; SL, spliced leader sequence; slRNA-seq, spliced-leader RNA sequencing.

Microarrays are dense molecular probe matrixes on a solid surface. DNA microarrays contain thousands of genes, gene fragments, and/or noncoding sequences that are hybridized with one or more labeled nucleic acid sample (DNA, cDNA, or RNA) for different purposes, such as gene expression profiling. In this case, total RNA or mRNA samples are directly labeled, amplified and labeled, or reversely transcribed in order to obtain directly or indirectly labeled cDNA. The fluorescent labels enable measuring the relative levels of each target sequence once emission signals have been acquired with a specialized scanner (Fig 2). Bioinformatics analysis is relatively simple because probes are usually identified beforehand, and just two basic steps are required: normalization and statistical analysis of differential gene expression (DGE). More technical details on microarray analysis can be found in reviews by Mantione and colleagues [62] and Lowe and colleagues [63]. A review of the DNA microarray technology impact in *Leishmania* research is also available [65].

RNA sequencing (RNA-seq) is a high-throughput approach based in Next Generation Sequencing (NGS) that consists of genome-scale amplification and NGS of short cDNA fragments generated from RNA samples. For this purpose, double-stranded cDNA is generated and PCR amplified, incorporating appropriate linkers for NGS. The primers used in all steps and the PCR conditions are designed according to the desired fragment size range, which is typically between 0.1 and 1 Kbp. The products are fragmented and subjected to NGS in any of the platforms commercially available (464-pyrosequencing, Illumina, Ion Torrent, etc.) (Fig 2). Alternatively, fragmented RNA can be used as the input in the library preparation protocol. Bioinformatics analysis is complex because reads of up to 300 bp [122] must be mapped on the genome sequence, which requires demanding skills. Further information on technical details has been reviewed by Mantione and colleagues [62] and Lowe and colleagues [63].

Nowadays, transcriptome analysis is a routine technical approach thanks to the development of the DNA microarray technology during the mid-1990s, which has been extensively used during the last 2 decades and is being replaced by RNA-seq. At this point, it is important to remark that the condition for a technical approach to be valid is reliability rather than novelty. Both DNA microarray hybridization analysis and RNA-seq are reliable for gene expression profiling or DGE analysis, although RNA-seq is a more powerful and robust approach [123, 124]. Microarrays and RNA-seq are technically reproducible (>99%) and accurate (approximately 90%) high-throughput approaches. Both can detect splice variants. However, RNA-seq requires much less input of RNA sample amount to reach the same genome coverage, is approximately 1,000 times more sensitive, and is characterized by lower background levels and a dynamic range approximately 100–1,000 times higher. In addition, RNA-seq is appropriate for SNP detection and UTR analysis and does not necessarily require a reference genome sequence [67, 123, 124].

Before execution of a DGE analysis, biological samples must be examined to determine whether they are appropriate to address the proposed hypothesis. For example, the main features of metacyclic promastigotes are high infectivity and morphology (fusiform, small size, showing an elongated flagellum). Therefore, metacyclic promastigotes can be identified for downstream DGE by infection experiments (Fig 1B and 1C) [7, 25, 26] or morphological features [125]. Promastigotes dedifferentiate once isolated because they are nondividing forms [14]. In fact, *Leishmania* spp. is adapted to respond very quickly to different environments [126]. Considering the replacement principle, experimentation with animals can be substituted by in vitro infection of established myeloid cell lines. Given the scarce number of promastigotes obtained from each sand fly, each sample should be composed of a mixture of promastigotes from different sand flies. A fraction of the sample should be immediately processed for RNA isolation upon extraction from the gut (e.g., lysed in Trizol reagent) and the remaining fraction used as soon as possible for the infection experiment [7, 25, 26]. In contrast with RNA-seq, which always includes a PCR amplification step, DGE based on the DNA microarray technology is not suitable for very-low-input samples unless RNA is amplified. Thanks to RNA amplification, as low as 20 ng of LiPro-Pper total RNA per replicate sample was sufficient to conduct transcriptome comparisons with intracellular amastigotes, stationary-phase promastigotes, and PNA^−^ promastigotes using microarray analysis [7, 25, 26]. Reliability of microarray results is not compromised by RNA amplification as otherwise suggested in [125]. In fact, reliability is improved regardless of whether it is required for sample expansion [127–129]. The amplification procedure consists of double-stranded cDNA synthesis starting from a poly(T) oligonucleotide incorporating the T7 promoter sequence upstream, followed by linear amplification by means of in vitro transcription (IVT) with the T7 RNA polymerase, obtaining reverse-complement RNA molecules ready for synthesis of labeled cDNA and subsequent hybridization with shotgun or oligonucleotide DNA microarrays. Preparation of RNA-seq libraries also requires synthesis of double-stranded cDNA and amplification, and the *L*. *major* RNA input was 5–20 ng [125]. The basic conceptual difference relies on PCR instead of IVT for required amplification for subsequent processing through high-throughput sequencing or labeled-cDNA synthesis and hybridization, respectively (Fig 2). Primer design is performed according to each high-throughput sequencing platform (e.g., Illumina adaptors and sequencing primers). Moreover, index sequences can be added for multiplexed sequencing. RNA-seq data analysis demands considerably more bioinformatics skills and computer resources than microarray analysis does [123].

The presence of tissue from the sand fly host should be minimized when isolating the biological sample. Microarray cross-hybridization controls were performed to select specific hybridization conditions and remove the few cross-hybridizing spots found from analysis [7, 25, 26]. Specific sequence alignment against the parasite's genome sequence would presumably remove most noise from sand fly sequences, but it may interfere in quantification of conserved sequences. Spliced-leader RNA-seq (slRNA-seq) is a fast, simple, and selective method that overcomes this inconvenience without biasing the results that would be obtained otherwise with a regular RNA-seq procedure [130, 131]. slRNA-seq allows for low input amounts of *L*. *donovani* RNA (1 ng) samples embedded in a human RNA amount 1,000 times larger, although these samples should be sequenced more deeply to reach the same coverage as pure *Leishmania* spp. RNA [130]. Once analysis is completed, validation of certain results by quantitative PCR (qPCR) or northern blot may be convenient. Even when the transcript levels have been validated, they do not quantitatively correlate to the protein levels in about 75% of cases [132]. Unfortunately, transcriptome analysis is the only feasible omics approach for sand fly–derived promastigotes so far because of the sample-amount requirements for translatome and proteome analysis (see "Translatome and proteome analysis: A major challenge" section). The number of qualitative RNA protein–level coincidences (up-regulation, down-regulation, and constant expression at both levels) in Lahav and colleagues’ [132] datasets is about 60%. This suggests that at least one-third of the changes in transcript levels will not be reflected in protein levels. Groups of functionally related genes showing transcript-level variation in the same sense (up-regulation or down-regulation) in the biological process under study will be more likely reflected at the protein level. This is also variable depending on the life cycle stages analyzed. For example, lower RNA–protein correlation has been observed across organisms under stress situations (fundamentally, the differentiation processes of procyclics to metacyclics and metacyclics to amastigotes) (reviewed in [133]). mRNA changes not correlated to protein levels may also be important for regulation of steady-state transcript levels. Mature RNA can be immediately used for protein synthesis or be stabilized and indefinitely kept translationally inactive (reviewed in [134]). Modulation of translational efficiency is an additional gene expression–regulation mechanism [135].

Four DGE analyses of *L*. *infantum* promastigotes obtained from experimentally infected *P*. *perniciosus* within the vector [7, 25, 26, 69] and one of *L*. *major* from *P*. *duboscqi* [125] have been performed (Table 1). An slRNA-seq analysis of heterogeneous populations has also been published [69]. The outcomes of these studies are considerably different fundamentally because the comparisons are not equivalent. First, *L*. *infantum* is responsible for zoonotic visceral leishmaniasis in the Mediterranean Basin and South America, whereas *L*. *major* is responsible for cutaneous leishmaniasis in the Old World. Their different affinity for sand fly vector species and in key developmental processes (e.g., attachment of nectomonads to the gut epithelium; see above) is a probable cause of obtaining mismatched DGE. Second, most samples and comparisons are not equivalent. For example, intracellular *L*. *infantum* amastigotes obtained in vitro from the myeloid human U937 cell line [26] are not equivalent to intracellular *L*. *major* amastigotes obtained from mice footpad lesions (LmAM) [125]. As it could be expected, the number of ≥2-fold differentially expressed genes (DEGs) was 2.4 times greater in the latter, where more complex biological samples represented not only the parasite and the host cell themselves but also the complex interactions with other immunological components. In both cases, DEG data referred to *L*. *infantum* (LiPro-Pper) and *L*. *major* sand fly metacyclic promastigotes (LmSFMP). In the first case [26], they were isolated from the anterior pole of the PSG plug in contact with the SV because this location is enriched in metacyclics, and their infectivity was checked by using the in vitro infection model (see above). Haptomonad promastigotes are also present in any residual material carried over from the SV structure (Fig 1B). In the second case, procyclics, nectomonads, and metacyclics were isolated from different guts and processed individually, assuming that the populations were homogeneous after 2, 4, and 15 days of development, respectively. The whole guts were macerated, promastigote populations were quantified with a hemocytometer, and morphology was examined. Only samples that were supposed to have >90% stage homogeneity were included for analysis [125]. However, squeezing whole guts does not necessarily guarantee homogeneity of populations, even when timing is expanded, because different parasite forms are always remaining in the gut. For example, Killick-Kendrick and colleagues [27] did not find more than 10% of *L*. *infantum* metacyclics in the *P*. *perniciosus* gut even 8–15 days after blood feeding from infected dogs. As mentioned above, this is dependent on the parasite–vector pair. In summary, all populations analyzed in the studies listed in Table 1 are homogeneous, with the exception of the study comparing heterogeneous populations on purpose [69]; but complete sample homogeneity is impossible to reach nowadays. An alternative analysis strategy is single-cell genomics. Unfortunately, molecular markers are not available for metacyclic promastigotes, which are the result of metacyclogenesis. HASP and SHERP are metacyclogenesis markers (i.e., they are expressed not only in metacyclic promastigotes but also in intermediate stages) in *L*. *major* [103]. For these reasons, comparisons of LiPro-Stat with LiPro-Pper and LiPro-PNA^−^ with LiPro-Pper [7, 25] are not equivalent to comparisons of LmSFMP with sand fly procyclics (LmSFPP) [125] or culture metacyclics (LmCMP) versus log phase promastigotes (LmPro-Log) [136]. For example, amino acid transporters aATP11 were up-regulated in LmSFMP versus LmSFPP and in nectomonad promastigotes (LmSFNP) versus LmSFPP [125], but it was not observed in LiPro-Pper versus LiPro-Stat, possibly because LiPro-Stat populations could contain nectomonad-, leptomonad-, and metacyclic-like forms [21]. Consistently, no aATP11 was differentially regulated when comparing LiPro-Pper and LiPro-Stat either [25]. Not only is the experimental design different in order to answer different biological questions but also the parasite–vector models are different in many instances. For example, only one kind of promastigote–sand fly gut interaction is clearly known so far, which is the LPG–galectin binding mechanism, only demonstrated in the *L*. *major*–*P*. *papatasi* and *L*. *major*–*P*. *duboscqi* pairs (reviewed in [23]). Another example is the gut microbiota, which has been shown to favor promastigote differentiation in *L*. *longipalpis* [137] but may be different in other sand fly species. In summary, generalization across *Leishmania*–sand fly models should be cautiously considered case by case, and different experimental settings should be taken into account when comparing DGE studies. An example of correct generalization is the HASP/SHERP cluster, gp63, and autophagy genes in *L*. *major* and *L*. *infantum* (see next section).

**Table 1:** Transcriptome studies and sample abbreviations.

Ref.	Stages	Microenvironment	Comparisons	Approach
[24]	PNA^+^ versus PNA^−^ Stat Pro	Culture	LiPro-PNA^+^ versus LiPro-PNA^−^	Microarrays
[25]	SV-derived versus PNA^−^ Pro	*P*. *perniciosus* gut versus culture	LiPro-Pper versus LiPro-PNA^−^	Microarrays
[7]	SV-derived versus Stat Pro	*P*. *perniciosus* gut versus culture	LiPro-Pper versus LiPro-Stat	Microarrays
[26]	SV-derived Pro versus Ama	*P*. *perniciosus* gut versus human cell line	LiPro-Pper versus LiAma	Microarrays
[125]	Nectomonad versus procyclic Pro	*P*. *duboscqi* gut	LmSFNP versus LmSFPP	RNA-seq
	Metacyclic versus procyclic Pro	*P*. *duboscqi* gut	LmSFMP versus LmSFPP	
	Ama versus procyclic/metacyclic Pro	*P*. *duboscqi* gut versus BALB/c mice footpad lesions	LmAM versus LmSFPP/LmSFMP	
[69]	All-gut versus culture forms	*P*. *perniciosus* whole-gut versus culture mixtures	LisfPro versus LiacPro	RNA-seq
[136]	Procyclic versus metacyclic Pro	Culture	LmCPP versus LmCMP	RNA-seq
[8]	Log versus Stat Pro	Culture	LiPro-Log versus LiPro-Stat	Microarrays

Original abbreviations have been used. Abbreviations: ac, mixture from axenic culture; Ama, amastigotes; CMP, culture metacyclic promastigotes; CPP, culture procyclic promastigotes; Li, *L*. *infantum*; Lm, *L*. *major*; Log, logarithmic phase; PNA, peanut agglutinin; Pper, *Phlebotomus perniciosus* stomodeal valve; Pro, promastigotes; Ref., reference; RNA-seq, RNA sequencing; sf, sand fly whole midgut; Stat, stationary phase; SFPP, sand fly procyclic promastigotes; SFMP, sand fly metacyclic promastigotes; SV, stomodeal valve.

The across-experiment comparison of LmSFMP/LmSFPP and LmCMP/LmPro-Log [125] is presumably robust even when the technical RNA-seq approach is not exactly the same, as supported by the methodological study on meta-analysis of RNA-seq expression data by Sudmant and colleagues [138]. Only 26 DEGs were claimed to differ between both datasets, but actually, the number of genes differentially expressed ≥2-fold at a statistical level of significance α = 0.05 is 398 in LmSFMP/LmSFPP [125] and only 108 in the case of LmCMP/LmPro-Log [136], of which 72 are not coincident. In the case of *L*. *infantum*, the number of DEGs found in the direct comparison of LiPro-Pper with LiPro-PNA^−^ was 285 at the cutoff expression values mentioned above [25], comparable to the number of LiPro-Pper/LiPro-Stat DEGs [7]. Most DEGs were different between both *L*. *infantum* datasets, which reflects the above-mentioned differences found in infectivity between these promastigote populations (LiPro-Pper > LiPro-PNA^−^ > LiPro-Stat). All *L*. *major* and *L*. *infantum* datasets are different because different stages have been compared in each case. For example, the LmSFMP/Lm-SFPP DGE analysis is not comparable to the LiPro-Pper/LiPro-Stat study because cultures in stationary phase mostly contain nectomonads and metacyclics [21] and probably low amounts of procyclics and leptomonads. In an slRNA-seq analysis of *L*. *infantum* comparing heterogeneous populations of sand fly promastigotes (LisfPro) [69] taken from the whole gut of *P*. *perniciosus*, with the heterogeneous promastigote populations in axenic culture (LiacPro), we observed approximately 950 genes up-regulated ≥2-fold, which is 2.0 to 3.6 times higher than expected compared with the previous DGE datasets about more homogeneous promastigote populations showing approximately 300 DEGs each [7, 25, 125]. Therefore, the DGE rates are, relatively, not very high in *Leishmania* spp., including homogeneous and heterogeneous populations (maximum approximately 1,000 DEGs out of approximately 8,300 genes annotated in the genome sequences). In summary, global concordances and differences between studies on sand fly–derived promastigotes have been found, but comparative interpretation of studies should be cautious, considering different biological comparisons, sample source origin and preparation, and technical approaches.

## What has transcriptome analysis taught?

The microenvironment influences the parasite’s differentiation processes [7, 125]. Steady-state transcript-level changes of the glucose-6-phosphate N-acetyltransferase, the cytochrome oxidase subunit VI, the vacuolar proton-translocating pyrophosphatase, and the amastin superfamily genes, when comparing promastigotes with amastigotes (all decreasing in amastigotes except for the amastins), were observed when promastigotes were obtained from the sand fly's SV [26] and from cultures [8]. However, most DEGs between LiPro-Stat and amastigotes are not coincident with DEGs between LiPro-Pper and amastigotes. Up-regulation of several amastin superfamily genes in metacyclics from the sand fly with respect to metacyclics from culture in *L*. *infantum* [25] and with respect to sand fly procyclics in *L*. *major* [125] provides additional evidence supporting the preadaptation hypothesis [8, 13, 26, 139–142], which consists of promastigote preparation in advance to survive within the host phagocytes (i.e., the amastigote stage). The highest levels of amastin transcripts are found in amastigotes when compared with both sand fly–derived promastigotes [26, 125] and cultured promastigotes [8].

Cell cycle–related genes are generally down-regulated in LmSFMP and LmSFNP compared with LmSFPP and LmAM, which is in agreement with the replicative or nonreplicative status of these stages [125]. Steady-state transcript–level comparisons between procyclic and metacyclic promastigotes in the sand fly gut (LmSFMP versus LmSFPP) [125] and in culture (LmCMP versus LmCPP) [136] resulted in relatively similar results because few differences were found between both studies. This includes transporters (pteridine transporter, nucleoside transporter 1, glucose transporters lmgt1 and lmgt2, amino acid transporters, and the ATP-binding cassette transporter ABC10), signaling molecules (phosphoprotein phosphatase and protein kinase LmjF.26.2570), calpain-like cysteine peptidase LmjF.30.2040, inosine guanosine nucleoside hydrolase, P27 protein, H2B and H4 histones, 4E-interacting protein LmjF.25.2450, the membrane-bound acid phosphatase 2 (MBAP2), and several hypothetical protein-encoding transcripts.

Many genes involved in metacyclogenesis (see below) are highly up-regulated in heterogeneous populations of sand fly-derived promastigotes (LisfPro) compared with cultured promastigotes (LiacPro) [69] but not in more homogeneous metacyclic populations (LiPro-Stat versus LiPro-Pper, LiPro-PNA^−^ versus LiPro-Pper, and LmSFMP/LmSFPP versus LmCMP/LmPro-Log) [7, 25, 125]. Comparing *L*. *infantum* heterogeneous populations composed of all promastigote development forms from the sand fly (whole-gut preparations) and culture (growth curve mixtures), we also observed that gp63 and autophagy genes were up-regulated [69], as well as the *HASP/SHERP* cluster. As mentioned above, these genes are essential for metacyclogenesis at least in *L*. *major*. In fact, Inbar and colleagues’ [125] results are in agreement because gp63 and autophagy gene up-regulation was found in LmSFNP. In addition, they found that *LPG3*, a gene essential for biosynthesis and assembly of GPI-anchored glycoconjugates, reaches its expression peak in LmSFPP. Sand fly–derived populations enriched in metacyclics (LiPro-Pper) are more infective than stationary-phase cultures (LiPro-Stat) and metacyclics obtained from those populations (LiPro-PNA^−^) [7, 25]. Autophagy, gp63, and *HASP/SHERP* gene cluster up-regulation in sand fly–derived promastigotes compared with cultured promastigotes supports that metacyclogenesis is more successful in the sand fly gut than in culture. Therefore, the microenvironment exerts an important influence in differentiation [7].

SHERP is essential for metacyclogenesis in *L*. *major* [103]. Inbar and colleagues [125] revealed evidence supporting this statement that consists of *SHERP* up-regulation in LmSFNP and LmSFMP, reaching maximum levels in LmSFMP. *L*. *infantum* transcriptome analysis is also in agreement with the role in metacyclogenesis, but *SHERP* transcripts are less abundant in LiPro-Pper than in LiPro-Stat [7], indicating that the levels are higher in nectomonads and leptomonads in culture (major forms within the stationary phase compared with metacyclics) than in sand fly–derived metacyclics. *SHERP* is not differentially expressed between LiPro-Pper and LiPro-PNA^−^, indicating that different microenvironments do no influence *SHERP* expression in *L*. *infantum* [25]. Stationary-phase promastigote cultures mostly contain nectomonad promastigotes [21], whereas most promastigotes derived from the sand fly’s SV and isolated using the PNA-negative selection method are metacyclic. *HASP-A1* is also down-regulated in LiPro-Pper versus LiPro-Stat [7], leading to the same conclusion about metacyclogenesis because this is also an essential gene for this process (see above). *L*. *infantum* sfPro versus acPro (heterogeneous populations) transcriptome analysis is also consistent with the previous studies because *SHERP* is up-regulated in sfPro (i.e., metacyclogenesis taking place more extensively in sand fly than in culture). Interspecies comparison should be cautious, as previously mentioned. *SHERP* data are concordant between *L*. *major* and *L*. *infantum* with the previously established idea about essentiality for metacyclogenesis, but simultaneously, transcriptome analysis has revealed specific differences.

Genes involved in fatty acid biosynthetic processes are up-regulated in sand fly–derived metacyclics in both *L*. *infantum* and *L*. *major* [7, 125], but the highest levels of these transcripts are reached in LmSFNP. According to DGE, glucose catabolism may be more pronounced not only in LmSFPP than in LmSFMP [125] but also in cultured than in sand fly–derived promastigotes (LiPro-Stat versus LiPro-Pper) [7]. Certain amino acid biosynthesis processes seem more active in culture according to DGE [7]. Genes involved in ATP synthesis–coupled proton transport are up-regulated in sand fly metacyclics (LiPro-Pper versus LiPro-Stat and LiPro-Pper versus LiPro-PNA^−^). According to relative infectivity (LiPro-Pper > LiPro-PNA^−^ > LiPro-Stat), sand fly metacyclics are “more metacyclic” than culture metacyclics. These findings are consistent with the considerable energy requirements for high motility ascribed to metacyclic promastigotes [14].

Confrontation of the transcriptomes and infectivity of sand fly–derived promastigotes with cultured promastigotes [7] is in agreement with the principle of nonequivalence of stationary-phase promastigotes supported by Gossage and colleagues [14]. Both transcriptomes showed moderate correlation in gene expression and 286 DEGs, and infectivity was approximately 30%–50% higher in LiPro-Pper. On the basis of these results, it was postulated that the adequacy of axenic promastigotes may depend on each particular experimental aim and design [7]. The characteristic transcriptome profiles found in LmSFPP, LmSFNP, and LmSFMP [38] are presumably a consequence of their adaptation to the different microenvironments in the vector as well. In fact, 72 out of the 108 DEGs found in LmCM/LmPro-Log [136] were not found among the 398 DEGs found in LmSFMP/LmSFPP, as stated above. Inbar and colleagues [125] performed LmSFMP versus LmSFPP differential expression analysis and compared data with an analogous experiment using cultured parasites (LmCMP versus LmCPP) [136]. Both studies were performed using the same RNA-seq procedure. These data are not comparable to LiPro-Pper versus LiPro-PNA^−^ promastigotes because this is a direct comparison [25] and these populations are not normalized to their initial procyclic promastigote forms. In other words, directly comparing sand fly–derived and culture-derived metacyclics does not correspond to comparing the differences between metacyclics and procyclics in both environments, unless procyclics from culture were exactly equal to procyclics in the sand fly, which is very unlikely. Different isolation methods may also influence the results (see the previous section).

A considerable number of the DEGs are involved in signal transduction and gene expression regulation at the posttranscriptional, translational, and posttranslational levels between cultured and sand fly–derived promastigotes [7, 25]. However, the biological implications of these findings remain unknown (see below). The finding that consists of translational efficiency being lower in differentiated nondividing metacyclic epimastigotes than in undifferentiated dividing *Trypanosoma cruzi* epimastigotes [143, 144] should guide interpretation.

Promastigotes constitutively secrete exosomes to the sand fly gut lumen. Coinoculation of cultured *L*. *major* promastigotes with sand fly gut–derived *L*. *major* exosomes leads to greater footpad lesions in mice [145]. These exosomes contain gp63 and other virulence factors [146–150]. These studies indicate that a parasite’s exosome content has immunomodulatory and signaling-inducing activities. Exosomes are secreted from multivesicular bodies (MVBs) and the flagellar pocket. Protein content of culture- and sand fly–derived promastigote exosomes is very similar [145]: gp63, which is secreted in the midgut and contributes to egestion [151]; HSP70 [152] and HSP83 [145]; calpain-like cysteine peptidases [153]; tryparedoxin peroxidase [154]; and surface antigen proteins [155]. Transcripts encoding for these proteins were also found increased in sfPro versus acPro [69].

## Unanswered questions about development and metacyclogenesis within the sand fly gut

Metacyclic promastigotes are defined by morphology, but their molecular features are not entirely known. PNA separation is effective to obtain highly infective promastigotes because PNA^−^ promastigotes are more infective than PNA^+^ in both *L*. *major* [22] and *L*. *infantum* [24], but the subpopulations obtained by this procedure may not be entirely equivalent in other species. A major LPG role in parasite–vector interaction is well defined only for *L*. *major*, whereas the parasite-interaction mechanisms remain unknown in all other species. LPG-independent promastigote development has been demonstrated in permissive vector species (see "Sand fly–*Leishmania* interactions" section). However, highly infective (therefore metacyclic) promastigotes isolated using the PNA-negative selection procedure is possible in *L*. *infantum* [24, 118, 156], which usually develops in permissive vectors such as *P*. *perniciosus*. Alternative unknown mechanisms participate in recognition because LPG is not strictly required for development, and the importance of this molecule is relegated to *L*. *major* only [31]. However, it is produced in all *Leishmania* species. Unknown PG receptors recognize the LPG in the sand fly gut [115, 117], which has at least an additional function acting as a shield against proteolytic activity during the first *L*. *major* development stages (see "Sand fly–*Leishmania* interactions" section), and presumably in *L*. *infantum* because both contain the key repeated (Gal-Man-PO_4_) motif in the LPG structure [114]. Variation of the LPG structure (see "The axenic culture model: Strengths and limitations" section) at the last stages toward the metacyclic stage makes negative selection with PNA possible in both species. Surprisingly, PNA^−^ and PNA^+^ subpopulations could be isolated in the monoxenous parasite *C*. *fasciculata* [121], a fact of unknown meaning suggesting that PG derivatives capable of agglutinating with the PNA may have more than one function. Studying LPG function in *C*. *fasciculata* may lead to raising other approaches for searching LPG interactions and alternative functions in different *Leishmania* species. High-throughput comparative metabolomics approaches may be useful to answer these questions, but not transcriptomics approaches. Bearing these considerations in mind, we suggest that the role of the modified LPG at this stage may not be necessarily the same between species, as already shown for the unmodified LPG at earlier stages. Consequently, we postulate that the “metacyclic status” of PNA^−^ from *L*. *infantum* may not be necessarily the same as for PNA^−^ from *L*. *major*, as the molecular markers and infection mechanisms may be different depending on the species. This is not surprising, because each species complex causes different pathology, and accurate measurements comparing metacyclic promastigote infectivity of each species are not possible. The peanut lectin has different affinity for LPG from a distinct origin, as different substitutions of the molecule disaccharide backbone are found depending on the species (see "The axenic culture model: Strengths and limitations" section). In any case, *L*. *major* [22] and *L*. *infantum* PNA^−^ promastigotes [24] have been demonstrated to be more infective than PNA^+^ promastigotes.

When comparing the heterogeneous populations LisfPro and LiacPro by slRNA-seq, a group of genes directly involved in metacyclogenesis was found to be highly up-regulated (≥4-fold) [69], which suggests that they are required during most stages of the developmental process within the sand fly gut compared with culture, not just at the last developmental stages. This includes five out of 14 autophagy genes, four out of eight gp63 genes, the HASP gene cluster (*HASPA1*, *HASPA2*, *HASPB*, respectively, LinJ.23.1200, LinJ.23.1220, and LinJ.23.1240), one out of three membrane-bound acid phosphatases (LinJ.28.2850), all three apical membrane antigen 1 (ama1, LinJ.30.1470, LinJ.30.1480, and LinJ.30.1490) proteins, and the META domain–containing protein 2 (*META2*, LinJ.17.0970) gene. Both small hydrophilic surface protein–encoding gene copies (*SHERP*, LinJ.23.1210, and LinJ.23.1230) are not included in the LisfPro versus LiacPro DEG set according to the 2-fold threshold value imposed, but they still show statistically significant approximately 1.5-fold higher levels in sfPro versus acPro [69]. Whereas *SHERP* is clearly up-regulated in *L*. *major* metacyclics (LmSFMP versus LmSFPP and LmCMP versus LmPro-Log) and, to a lower extent, in nectomonads (LmSFNP versus LmSFPP) [125, 136], different expression profiles supporting an overexpression maximum in nectomonads (LiPro-Pper versus LiPro-Stat) [7] (see the reasons in the previous section) were observed in *L*. *infantum*. Although the specific *SHERP* expression profiles are different, both are concordant with SHERP essentiality in metacyclogenesis [103]. Cultured and sand fly–derived *L*. *infantum* and *L*. *major* metacyclics differentially regulate *SHERP* expression (LiPro-Pper versus LiPro-PNA^−^, and comparison between LmSFMP versus LmSFPP and LmCMP versus LmCPP). Interestingly, both *SHERP* genes are up-regulated in LiPro-Stat versus LiPro-Log of this species according to microarray analysis [8] and further confirmation by qPCR in two independent works [24, 157]. This is equivalent to stating that the set of nectomonads, leptomonads, and metacyclics up-regulate *SHERP* compared with procyclics. *SHERP* is a good metacyclogenesis marker but not a metacyclics marker because it is overexpressed in more than one promastigote form (nectomonads and metacyclics). The data suggest that the *SHERP* gene expression patterns are similar between *L*. *major* and *L*. *infantum*, except for the promastigote form reaching the maximum expression levels, which peak earlier in *L*. *infantum* than in *L*. *major*. This would not be surprising whenever confirmed in the future given the different biological affinity for vectors and different developmental processes of both species, resulting in different disease progression in mammalian hosts. These observations are in agreement with the fact that metacyclic promastigote features and behavior may vary between species and are not entirely known. For example, they are highly infective, or more infective than other promastigote forms, but by how much? Which molecules are true markers of metacyclics in each species?

The *META1* gene was described to be expressed specifically at the metacyclic stage in culture, but the high-throughput DGE studies of *L*. *infantum* and *L*. *major* have not confirmed this result at the transcript level in sand fly–derived promastigotes [7, 25, 125]. As mentioned before and discussed below, studies at the protein level like western blot or proteomic approaches are not viable so far. About half of the genes annotated in the *Leishmania* spp. genomes encode for hypothetical proteins, most of unknown biological role in the parasite. These observations provide an idea of how little is known about development within the sand fly vector.

Elucidation of processes involving the unknown relationship between external stimuli from the microenvironment, the parasite’s uncharacterized sensing and intracellular signaling mechanisms, and the unusual gene expression regulation mechanisms found in these organisms (reviewed in [134, 158]) may probably help to further illustrate promastigote development within the sand fly gut. For these purposes, elucidation of signal transduction pathways and the underlying mechanisms affecting gene expression regulation is essential because more crucial genes in development may be found.

## Translatome and proteome analysis: A major challenge

In an experiment combining DGE analysis by means of DNA microarrays and quantitative proteomics with polysome profiling in *L*. *donovani*, Lahav and colleagues [132] observed that gene expression regulation is performed at the posttranscriptional, translational, and posttranslational levels, leading to find that only 25% of transcript levels were quantitatively correlated with the corresponding protein levels, as mentioned previously. Therefore, DGE at the translational and posttranslational levels is more directly related to physiological changes of the different life cycle stages than at the posttranscriptional level. A complete picture of DGE would be provided by combined transcriptome, translatome, and proteome analysis. Polysome profiling is an approach for measuring translational efficiency that consists of separation of mRNA–ribosome complexes (polysomes) according to their molecular weight by means of density gradient centrifugation for subsequent quantification of the fractions and high-throughput analysis of the mRNA molecules in each fraction. The procedure requires approximately 4 × 10^8^ cells (50 mL at an optical density of OD_600 nm_ = 0.6) in the case of *Saccharomyces cerevisiae* [159]. As the average cell volume of this yeast species is approximately 900 μm^3^ and the average volume of a *Leishmania* spp. cell is approximately 65–75 μm^3^, about 10 times more promastigotes or amastigotes would be required in principle.

Ribosome profiling is a more specific high-throughput approach for measurement of translational efficiency. Protection of mRNA sequences by ribosomes is quantified by means of NGS from a ribosome-footprinting library combined with a fragmented-mRNA library [160]. The first ribosome-profiling studies in trypanosomatids have revealed that changes in protein production between slender bloodstream and procyclic stages of *T*. *brucei* are more extensive than indicated by transcriptome profiling [135, 161]. In these approaches, at least 10^9^ parasites per sample were used to generate the ribosome-footprinting and the fragmented-mRNA library. Jensen and colleagues [135] also mapped the 5′ ends of mRNAs by means of slRNA-seq. The same general finding was reported for *T*. *cruzi* [144], in which a higher amount of parasites was used. Consequently, ribosome profiling is not viable for studies in *Leishmania* spp. promastigotes obtained from the sand fly so far. In fact, as many as approximately 10^4^ infected sand flies would be required to obtain enough promastigotes for a replicate of a ribosome-profiling experiment, and many more sand flies would be required for ribosome profiling of more homogeneous populations—for example, approximately 10^6^ for metacyclics.

Typical samples for proteome analysis require approximately 1–2 × 10^8^ *Leishmania* spp. cells for both two-dimensional electrophoresis-based strategies [162] and quantitative proteomics strategies [163]. Although this is about one-tenth to one-fifth of the amounts required for translatome analysis, the numbers still indicate that proteome analysis is not possible for sand fly–derived promastigotes either. Even western blot semiquantitative analysis of single-protein levels has not been tested so far and would be very challenging, if not impossible. Despite the approach being very sensitive, the challenge is to obtain sufficient sample and equalize amounts across samples in order to make them comparable. Consequently, only transcript levels can be analyzed so far. Although transcriptome analysis is very informative and many strategies based on this approach can be developed (e.g., DGE of knock-out or knock-in promastigote cell lines within the sand fly vector) leading to significant biological findings, the absence of low-input translatome and proteome approaches implies that many physiological aspects of promastigote development within the sand fly gut will remain unexplored for a long time.

## Concluding remarks

Metacyclic promastigotes are distinguished by morphology (rapid-swimming forms with an elongated flagellum) and high infectivity. No molecular markers are available. Metacyclics can be isolated by negative selection with PNA, as confirmed by infection experiments. Caution should be exercised when using cultured promastigotes depending on the experimental design, and when comparing studies. Transcriptome analysis has revealed the crucial microenvironmental role in parasite development in the sand fly gut because substantial differences and moderate correlation between cultured and sand fly–derived promastigotes have been found. In fact, sand fly–derived metacyclics are more infective than metacyclics in culture, and genes involved in metacyclogenesis such as the *HASP/SHERP* cluster, the *gp63* metalloprotease family, and autophagy genes are overexpressed in sand fly metacyclic promastigotes compared with cultured promastigotes. Differential expression of several genes involved in gene expression regulation, signaling, and metabolic processes between sand fly–derived and cultured promastigotes supports an important microenvironmental influence differentiation. Elucidating signal transduction pathways in these parasites may substantially improve understanding of the relationships between promastigotes and the different microenvironments in the sand fly gut (Table 2). Unfortunately, translatome and proteome analysis is not feasible in promastigotes obtained from the sand fly gut so far.

**Table 2:** Functional genomics in sand fly–derived promastigotes: Main findings.

Ref.	Main findings
[7, 125]	The microenvironment influences parasite differentiation.
[7, 25]	Sand fly–derived promastigotes from the stomodeal valve are more infective than stationary-phase and PNA^−^ cultured promastigotes. Approximately 300 genes are differentially regulated.
[69, 125]	Autophagy, gp63, and HASP/SHERP cluster genes are up-regulated during metacyclogenesis (nectomonad and metacyclic promastigotes). These findings confirm that these genes are metacyclogenesis markers.
[125]	Pteridine, glucose, nucleoside, and amino acid transporter genes are up-regulated in *L*. *major* sand fly–derived versus cultured metacyclics.
[125]	Calpain-like cysteine peptidase, membrane-bound acid phosphatase 2, and several signaling molecule–encoding genes are up-regulated in *L*. *major* sand fly–derived versus cultured metacyclics.
[7, 25, 26]	Many signal transduction genes are differentially expressed between cultured and sand fly–derived promastigotes.
[7, 25, 26, 66]	Most signal transduction mechanisms are unknown in *Leishmania* parasites. Therefore, changes between sand fly–and culture-derived promastigotes are unknown.
[69, 125]	Several genes involved in fatty acid biosynthetic processes are up-regulated in sand fly–derived *L*. *major* and *L*. *infantum* promastigotes.
[145]	Promastigotes secrete exosomes to the sand fly gut lumen. Coinoculation with *L*. *major* promastigotes leads to magnified footpad lesions in mice.
[145]	Protein content of culture- and sand fly–derived promastigote exosomes is very similar.
[146–150]	gp63 and other virulence factors are present in exosomes.
[69, 145, 151–155]	Several proteins contained in promastigote exosomes (gp63, HSP70, HSP83, calpain-like cysteine peptidases, surface antigen proteins, etc.) are up-regulated in whole-gut sand fly–derived promastigotes.

Abbreviations: gp63, glycoprotein 63; HASP, hydrophilic acidic surface protein; PNA, peanut agglutinin; Ref., reference; SHERP, small endoplasmic reticulum protein.

The main outstanding questions are: (1) What are the molecular features of the different *Leishmania* spp. promastigote forms? (2) Are the multiple roles of the LPG different between species, causing different types of leishmaniasis? (3) Are there truly stage-specific markers? (4) Are they different between species? (5) How different are canonical signal transduction cascades and those of *Leishmania* spp.? (6) Are there developmentally regulated changes in Trans-splicing? If so, what implications would they have? (7) How can relative protein levels be analyzed in sand fly–derived promastigotes?

**Key learning points**

- Metacyclic promastigotes are highly infective forms, but no markers are available.
- Accurate description of samples compared by means of high-throughput strategies and caution when comparing different studies are essential and are particularly important for samples obtained from the sand fly because different vector and parasite pairs are considered.
- Transcriptome data and infection experiments support that sand fly–derived promastigotes are more infective than cultured ones.
- Sand fly–derived promastigotes are more infective than cultured promastigotes to in vitro–cultured human phagocytes, which combined with transcriptome profiles, supports that metacyclogenesis is more successfully completed in the sand fly gut.
- Transcriptome analysis in *Leishmania infantum* and *L*. *major* promastigotes derived from the sand fly gut confirm that the hydrophilic acidic surface protein (*HASP*), the small hydrophilic endoplasmic reticulum protein *SHERP*, and the glycoprotein 63 (*gp63*) genes are involved in metacyclogenesis and already increased in nectomonad promastigotes and thus are not metacyclic promastigote markers.
- Differential expression of several genes involved in gene expression regulation, signaling, and metabolic processes between sand fly–derived and cultured promastigotes supports an important influence of the microenvironment in differentiation.
- Studying the translatome and the proteome is not feasible in sand fly–derived promastigotes so far. Transcriptomics is the only alternative, and interpretation of the results should be cautiously discussed because transcript levels do not always reflect protein levels.

**Top five papers**

1. Alcolea PJ, Alonso A, Gomez MJ, Postigo M, Molina R, et al. Stage-specific differential gene expression in Leishmania infantum: from the foregut of Phlebotomus perniciosus to the human phagocyte. BMC Genomics. 2014;15:849.
2. Alcolea PJ, Alonso A, Dominguez M, Parro V, Jimenez M, et al. Influence of the microenvironment in the transcriptome of Leishmania infantum promastigotes: Sand fly versus culture. PLoS Negl Trop Dis. 2016;10(5):e0004693.
3. Alcolea PJ, Alonso A, Degayon MA, Moreno-Paz M, Jimenez M, et al. In vitro infectivity and differential gene expression of Leishmania infantum metacyclic promastigotes: negative selection with peanut agglutinin in culture versus isolation from the stomodeal valve of Phlebotomus perniciosus. BMC Genomics. 2016;17:375.
4. Alcolea PJ, Alonso A, Baugh L, Paisie C, Ramasamy G, Sekar A, et al. RNA-seq analysis reveals differences in transcript abundance between cultured and sand fly-derived Leishmania infantum promastigotes. Parasitol Int. 2018;67(4):476–80.
5. Inbar E, Hughitt VK, Dillon LA, Ghosh K, El-Sayed NM, et al. The transcriptome of Leishmania major developmental stages in their natural sand fly vector. MBio 2017;8(2):e00029-17.
